# Overexpression of Glyoxalase 2 in Human Breast Cancer Cells: Implications for Cell Proliferation and Doxorubicin Resistance

**DOI:** 10.3390/ijms252010888

**Published:** 2024-10-10

**Authors:** Brenda Romaldi, Andrea Scirè, Cristina Minnelli, Andrea Frontini, Giulia Casari, Laura Cianfruglia, Giovanna Mobbili, Lidia de Bari, Cinzia Antognelli, Federico V. Pallardó, Tatiana Armeni

**Affiliations:** 1 Department of Odontostomatologic and Specialized Clinical Sciences, Università Politecnica delle Marche, 60131 Ancona, Italy; b.romaldi@pm.univpm.it (B.R.); g.casari@staff.univpm.it (G.C.); lauretta2086@gmail.com (L.C.); 2 Department of Life and Environmental Sciences, Università Politecnica delle Marche, 60131 Ancona, Italy; a.a.scire@staff.univpm.it (A.S.); c.minnelli@staff.univpm.it (C.M.); a.frontini@staff.univpm.it (A.F.); g.mobbili@staff.univpm.it (G.M.); 3 Institute of Biomembranes, Bioenergetics and Molecular Biotechnologies (IBIOM), National Research Council (CNR), 70126 Bari, Italy; l.debari@ibiom.cnr.it; 4 Department of Medicine and Surgery, Università degli Studi di Perugia, 06129 Perugia, Italy; cinzia.antognelli@unipg.it; 5 Department of Physiology, Medicine and Dentistry School, University of Valencia-INCLIVA, Center for Biomedical Network Research on Rare Diseases (CIBERER), 46010 Valencia, Spain; federico.v.pallardo@uv.es

**Keywords:** glyoxalase 2 (Glo2), breast cancer cells, nucleus, proliferation, S-glutathionylation, redox metabolism

## Abstract

Glyoxalase 2 (Glo2) is an enzyme of the glyoxalase system whose pathway parallels glycolysis and which aims to remove methylglyoxal (MGO). This study analyzed the possible additional roles of the Glo2 enzyme in breast cancer (MCF7) and non-cancer (HDF) cell lines, investigating its presence at the nuclear level and its potential involvement in cell proliferation and chemotherapy resistance. The results revealed that Glo2 is overexpressed in cancer cells, and its expression is higher during the proliferative (S and G2/M) phases of the cell cycle. The study also examined a post-translational modification (PTM) in which Glo2 could be involved, with S-glutathionylation revealing that Glo2 enhances this PTM in cancer cells both in the cytoplasm and nucleus. Inhibition of Glo2 by p-NCBG resulted in increased sensitivity to doxorubicin, a common chemotherapeutic agent. This suggests that Glo2 increases cancer cell resistance to chemotherapy, potentially through its role in regulating oxidative stress. These results highlight Glo2 as a potential therapeutic target to improve the efficacy of existing treatments.

## 1. Introduction

The glyoxalase system is an important glutathione (GSH)-dependent pathway whose major role in all living organisms is the detoxification of methylglyoxal (MGO), a by-product of cell metabolism, especially glycolysis [1]. The glyoxalase system consists of glyoxalase 1 (Glo1), which catalyzes, using GSH as a cofactor, the isomerization of MGO into S-D-lactoylglutathione (SLG), and glyoxalase 2 (Glo2), which catalyzes the hydrolysis of SLG into D-lactate, releasing GSH [2,3]. Generally, both Glo1 and Glo2 are overexpressed in cancer cells as there is a need to rapidly metabolize the cytotoxic MGO that continuously accumulates because of the elevated glycolytic flux and metabolic activity of cancer cells [4,5]. Although traditionally known as a thiolesterase, Glo2 has been recently described as also having non-enzymatic functions [6]. For instance, it was found that Glo2 can induce post-translational modifications (PTMs) to target proteins due to N-acetylation of the ε-amino of lysine residues of mitochondrial proteins and S-glutathionylation of the sulfhydryl groups of cysteine residues of some proteins [7,8,9,10]. Therefore, Glo2 is able to interact with other proteins, such as actin, malate dehydrogenase (MDH) and the pro-apoptotic protein Bax, thus, suggesting additional roles for this protein in specific biological responses and cellular pathways [11,12].

Indeed, in MCF7 breast cancer cell lines it has been shown that cytosolic Glo2, by inhibiting apoptosis, acts as pro-survival factor for the p53 family [13]. Another study in prostate cancer has shown that Glo2 promotes cell proliferation and apoptosis evasion through a mechanism involving the androgen receptor and the p53-p21 axis [14]. Moreover, in prostate cancer cell lines, it has been shown that a loss of PTEN induces activation of PTEN/PI3K/AKT/mTOR, which, in turn, activates the p-PKM2/Erα axis, leading to the up-regulation of Glo2 that is associated with an increase in cell survival, proliferation and cell migration [15]. On the other hand, Glo2, specifically the mitochondrial isoform, appears to be linked to the promotion of mitochondrial apoptosis in human non-small cell lung cancer (NSCLC) A549 cells, upon oleuropein exposure, through a pathway involving multiple steps and inducing the upregulation of mitochondrial Glo2, which interacts with the pro-apoptotic protein Bax, thus, activating apoptosis [12]. In zebrafish, Glo2 has been identified as a regulator of cellular energy metabolism and P70-S6 kinase activity [16]. Finally, in Parkinson’s disease, Glo2 stabilizes mitochondria during cellular stress, supporting the survival of neurons [17], while in mice it contributes to supplying mitochondrial GSH levels [18]. Despite these multiple roles identified for Glo2, studies regarding the role of Glo2 in cancer cells are currently still very limited.

In this study, we investigated the possible presence of Glo2 at the level of the nucleus, although only two isoforms were identified for human Glo2, the cytosolic and the mitochondrial ones, which in vertebrates are encoded by a single gene, HAGH (hydroxyacylglutathione hydrolase) [19,20,21]. This gene, located on chromosome 16, gives rise to two distinct mRNA species transcribed from 9 and 10 exons, respectively. The mitochondrial form is encoded from the transcript derived from the nine exons, but this transcript also encodes the cytosolic form as it contains a downstream start codon. On the contrary, the ten exons’ transcript only encodes for the cytosolic form because it has an in-frame termination codon [22]. The molecular mass of the cytosolic form is around 29 kDa, while that of the mitochondrial one is approximately 34 kDa. The different molecular masses of the two isoforms are due to the absence of the first 48 amino acids in the cytosolic sequence, amino acids in which a mitochondrial targeting sequence (MTS) is contained [22,23,24]. As a result of this information, the presence of nuclear Glo2 could, potentially, come from the cytosol or mitochondria. In this paper, considering the multiple aspects of Glo2, we wished to establish its possible presence in the nucleus of breast cancer cells (MCF7s), as compared to normal fibroblast-derived cells (HDFs), to assess its possible involvement in cancer cell proliferation, and we also aimed to evaluate the effect of Glo2 inhibition alone or in combination with doxorubicin.

## 2. Results

### 2.1. Glo2 Is Strongly Detected in the Nucleus of Breast Cancer Cells

Because Glo2 enzyme activity has frequently been detected at higher levels in cancer cells than in non-cancer cells, in this study, we hypothesize that it may play a crucial role in regulating cell proliferation within the nucleus.

**Figure 1:**
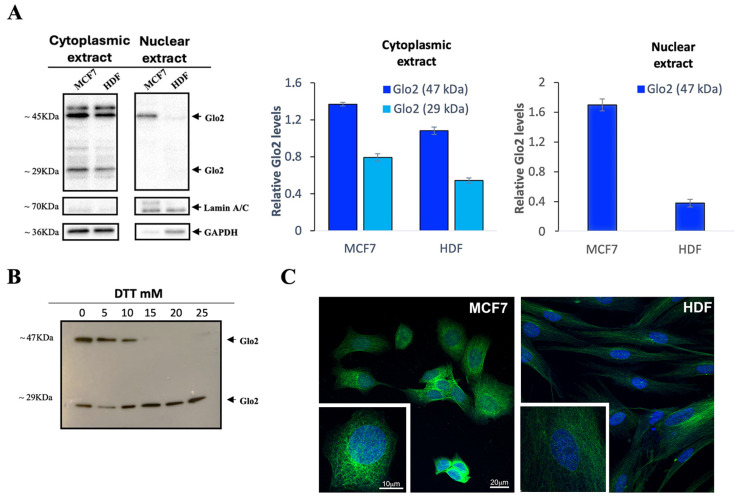
Localization of Glyoxalase 2 (Glo2) in the cytoplasmic and nuclear extracts of MCF7 and HDF cells. (**A**) Western blotting analysis of Glo2 in cytoplasmic and nuclear extracts of MCF7 and HDF cells. Alongside this, there is the densitometric immunoblot quantification for Glo2 protein. Cytoplasmic extracts show a 29 kDa and a 47 kDa band, while nuclear extracts show only the 47 kDa one, which is significantly more marked in MCF7 cells. Results are reported as mean ± S.D. of at least *n* = 6 different experiments. (**B**) MCF7 cytoplasmic extract treated with increasing concentrations of DTT, showing the disappearance of the 47 kDa band at the highest concentrations of DTT. (**C**) Representative images by immunofluorescence and confocal microscopy analysis of Glo2 (green) in permeabilized MCF7 and HDF cells (nuclei are visualized in blue using DAPI). The staining disclosed a clear localization of Glo2 in the nucleus and in the cytoplasm of both cell types. In the magnified inset, the resulting pattern of staining was more evident in the nuclear compartment and in association with the cytoskeleton of MCF7 cells. Scale bars in MCF7 panels apply also to the corresponding HDF panels.

First, we aimed to explore whether Glo2 was present in the nucleus of the cells, so we performed a Western blot analysis with anti-Glo2 antibody on cytoplasmic and nuclear extracts of human breast cancer cells (MCF7s) and normal fibroblast-derived cells (HDFs). In the cytoplasmic extracts, two bands of different molecular weights are evident: one at 29 kDa and another at approximately 47 kDa in both cell lines (Figure 1A). The 29 kDa band in the MCF7 cytoplasmic extract had approximately slightly less than double the intensity of the HDF one, while the 47 kDa band was slightly more pronounced in MCF7s than in HDFs. In the nuclear extracts, only the 47 kDa band was visible, and was markedly more intense in the MCF7 one as compared to the barely visible band in the HDF one. For this band, densitometric analysis indicated a 6-fold higher intensity in the MCF7 than in the HDF nuclear extract (Figure 1A, right panel). Instead, the nuclear extracts do not show the 29 kDa band, so only the bars for the 47 kDa band are present in the quantification graph. We cannot rule out with certainty the presence of the 29 kDa band in the nuclear extract because, although rarely, in the experimental replicates, a slight band could also have been present in relation to the replicative timing of the cell population.

Since the canonical weight of Glo2 corresponds to 29 kDa, to exclude the possibility that the 47 kDa band was not an aspecific band, an additional experiment was performed. Specifically, the anti-Glo2 antibody was blocked by recombinant Glo2, and Western blotting was performed under this condition. The analysis, revealing no bands at 29 and 47 kDa, showed that these two bands were specific for the anti-Glo2 antibody (Figure S1).

In a subsequent experiment (Figure 1B), cytosolic extracts of MCF7 cells were treated with increasing concentrations of dithiothreitol (DTT) to further denature the disulfide bonds and evaluate the possible dissociation of the 47 kDa band. The results show that at the highest concentrations (15, 20 and 25 mM) of DTT, the 47 kDa band is not visible, leaving only the 29 kDa band, which corresponds to the molecular weight of the cytosolic isoform Glo2 (Figure 1B).

To additionally confirm the presence of Glo2 in the nucleus, IF and confocal microscopy analyses were conducted on MCF7 and HDF cells (Figure 1C). The images shown in Figure 1C were obtained by a single optical plan of about 0.5 µm thickness within the nuclear compartment. In this location, the staining showed a spot-like appearance within the nuclear compartment. The staining is also clear in the cytoplasm and the pattern of staining suggests an association with the cytoskeleton both in the HDF and MCF7 cells. The intensity of the staining was higher in the MCF7 cells compared to the HDF cells both at the cytoplasmic and nuclear levels. We have also collected z-stack images of selected cells to give an overall view of the nucleus and cytoplasm and to visualize potential differences in the compartmentalization of the staining. The Z-stack images displayed a consistent pattern of staining within the cells, indicating a homogeneous distribution in both the nucleus and cytoplasm.

### 2.2. Nuclear Glo2 Interacts at the Chromatin Level in MCF7 Breast Cancer Cells

**Figure 2:**
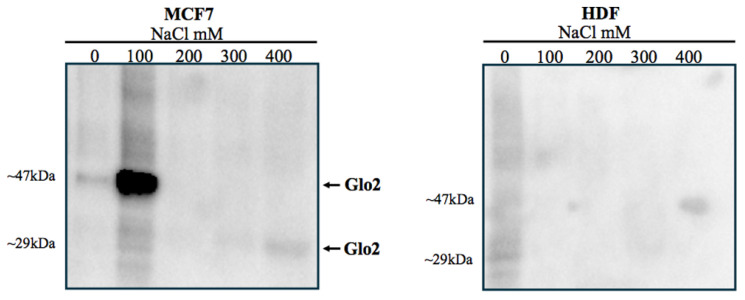
Sequential salt extraction on nuclei of MCF7 and HDF cells and immunoblot of Glyoxalase 2 (Glo2). Immunoblots of Glo2 elution profile were obtained from isolated nuclei of MCF7 and HDF cells treated with increasing NaCl concentrations (0–400 mM). It is evident (left membrane) that there is a spot at 47 kDa after extraction with 100 mM NaCl, while a weak band at 29 kDa is present after extraction with 400 mM NaCl. In HDF cells (right membrane), there are no visible bands, confirming the presence, to a much lesser extent, of Glo2 in the nucleus of non-cancerous cells. Molecular weights of markers are showed in Figure S6.

Once we established the presence of Glo2 in the nucleus of MCF7s, we aimed to assess what level of interaction Glo2 posed toward nuclear structures. Using the technique of sequential salt passages, we obtained increasingly purified nuclear extracts until we reached only DNA. This allowed the elution of specific proteins from bulk chromatin to be profiled. The analysis revealed, only in MCF7 cells, a well-determined spot at 47 kDa after extraction at 100 mM NaCl (Figure 2, left panel). The band has a weight that corresponds to that always evidenced in nuclear extracts of cancer cells. A weak band at 29 kDa can also be evidenced after treatment with 400 mM NaCl, suggesting that Glo2 as a protein in monomeric form, although in very small amounts, may be localized closer to the DNA helix than Glo2 at 47 kDa. In contrast, extraction at different NaCl concentrations in HDF cells shows no visible bands, confirming the presence, to a much lesser extent, of Glo2 in the nucleus of non-cancerous cells (Figure 2, right panel).

### 2.3. Glo2 in Different Phases of the Cell Cycle

To correlate Glo2 with cell cycle phases, we synchronized cells by culturing them in DMEM with 0.1% FBS for 48 h, then we performed immunoblot and enzyme activity analyses from cell extracts at time 0 (non-proliferating cells) and 48 h after reactivation with 15% FBS (proliferating cells) [25].

The data obtained after cell synchronization are shown in Table 1. At time 0 (non-proliferative phase), both cell lines showed an increased percentage of cells in the G0/G1 phase. Instead, 48 h after reactivation, both cell lines, especially the MCF7 cells, displayed an increased cell percentage in the G2/M phase. Cytograms are shown in the supplementary (Figure S2).

**Figure 3:**
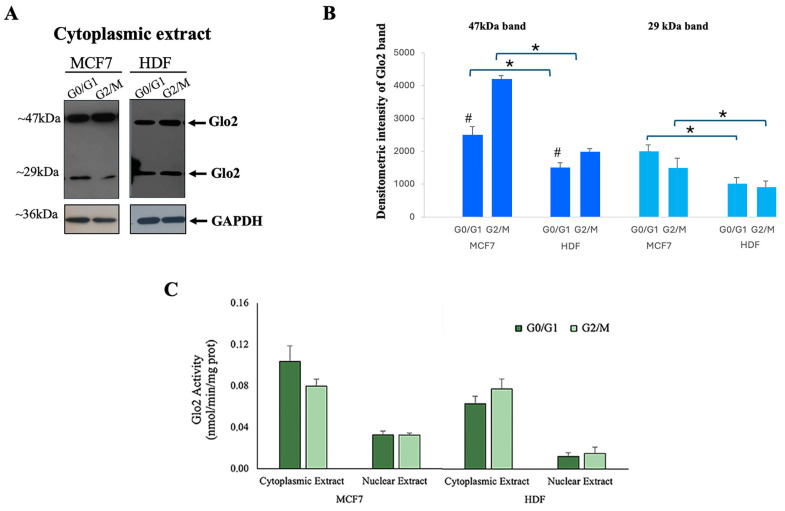
Localization of Glyoxalase 2 (Glo2) in different phases of cell cycle. (**A**) Western blotting with anti-Glo2 antibody of MCF7 and HDF cytoplasmic extracts in G0/G1 phase and G2/M phase, together with (**B**) densitometric immunoblot quantification. Asterisk represents the statistical analysis between MCF7s and HDFs, while gate symbols represent the statistical comparison between G0/G1 and G2/M phases, * *p* ≤ 0.05 and ^#^ *p* ≤ 0.01. (**C**) Glo2 enzymatic activity in cytoplasmic and nuclear extracts of MCF7 and HDF cells in G0/G1 and G2/M phases. Activity is given as nmol/min/mg proteins. Results are reported as mean ± S.D. of at least *n* = 4 different experiments. *p* > 0.05.

Figure 3A shows the results obtained by immunoblotting detection of Glo2 in cytoplasmic extracts of HDF and MCF7 cells in the G0/G1 phase (non-proliferating cells) and G2/M phase (proliferating cells). To the right of the blot, the densitometry quantification is represented (Figure 3B). In the cytoplasmic extract, two different weight bands (29 and 47 kDa) are evident. Cytoplasmic 47 kDa Glo2 protein is significantly higher in both MCF7 and HDF cells during the G2/M cell phases compared to the G0/G1 phases, and this difference is more evident for MCF7 cells. In general, a significantly greater presence of Glo2 in multimeric (47 kDa) form is evident in the G2/M phases in comparison to the G0/G1 phases. The enzymatic activity of Glo2 measured in the cytosol and nuclear extracts at different proliferative stages of HDF and MCF7 cells shows no significant differences (Figure 3C).

### 2.4. Efficacy Test of p-NCBG Inhibitor and Cytotoxicity of Glo2 Inhibition in HDF and MCF7 Cells

To evaluate the effect of Glo2 inhibition on the cell cycle, we pharmacologically inhibited Glo2 by using p-nitrocarbobenzoxyglutathione (p-NCBG), which specifically inhibits mammalian Glo2 without affecting Glo1. Table 2 shows the enzymatic activity of both enzymes. In the enzyme assay, recombinant Glo2 protein was added and the activity evaluated spectrophotometrically in the presence of increasing concentrations of p-NCBG inhibitor. The effectiveness of the inhibitor is demonstrated by the observation that already at 9 µM p-NCBG the enzymatic Glo2 activity is almost completely inhibited. Enzymatic Glo1 activity was also measured in the presence of the p-NCBG inhibitor to demonstrate the specificity of the inhibitor toward Glo2.

To evaluate the effectiveness of the inhibitor on cells, we treated MCF7 cells with p-NCBG inhibitor and we observed that the administration of free p-NCBG was not effective in inhibiting cellular Glo2 (Figure S3), probably due to its inability to pass through the cell membrane. Therefore, to improve its chemical stability in an extracellular medium and facilitate membrane transition, we encapsulated p-NCBG in liposomes (Lip-NCBG) made of POPC lipid. In this condition, we obtained a dose-dependent inhibition of Glo2 activity in MCF7 cells (Supplementary Figure S5) [26], highlighting the importance of drug delivery systems in improving the efficacy of a drug. Additionally, before performing the cell cycle experiment, preliminary studies were performed to select the safest dose of Lip-NCBG to use (Supplementary Figure S5) that was capable of inducing at least 60% inhibition of Glo2 activity. Based on these results, cells were pre-treated with liposome (Lip), which was used as a control, and Lip-NCBG for 48 h at final concentrations of 0.28 and 0.25 mM of liposome and p-NCBG, respectively (Supplementary Figure S4A). Moreover, to also assess the safety of Lip-NCBG in the normal cell line, we evaluated the cell viability in the HDF cell line. As for the MCF7 cells, treatment with Lip loaded with 0.25 mM p-NCBG did not induce any cytotoxic effect (Supplementary Figure S4B).

### 2.5. Lip-NCBG Affects the Cell Cycle Progression in Breast Cancer Cells

**Figure 4:**
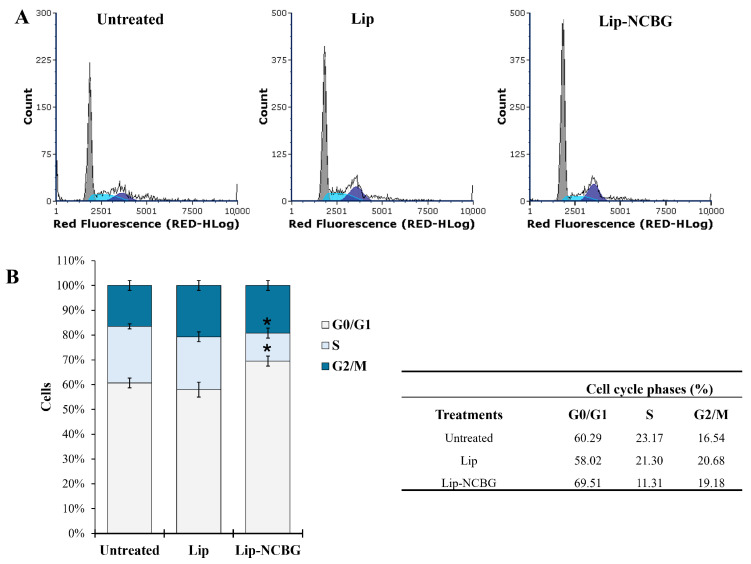
Effect of Glyoxalase 2 (Glo2) inhibition on cell cycle progression. (**A**) Representative cytograms of the cell cycle of synchronized MCF7 cells (not treated with Lip or Lip-NCBG) (left panel) and reactivated MCF7 cells treated with Lip (central panel) or Lip-NCBG (right panel) using propidium iodide (PI) (40 μg/mL) as probe. (**B**) Percentage of untreated, Lip treated and Lip-NCBG treated MCF7 cells in the various steps of the cell cycle. Table (right panel) of data obtained in the various phases of the cell cycle of untreated, Lip treated and Lip-NCBG treated MCF7 cells. * *p* < 0.05 with respect to Lip treated cells.

The cell cycle experiment is showed in Figure 4. MCF7 cells (named as untreated) were placed without FBS serum for 72 h to synchronize them, then reactivated with the addition of 15% FBS for 4 h and then treated with empty liposome or Lip-NCBG inhibitor for 32 h. The safer and more effective concentration of Lip-NCBG was used, with the empty liposome being used as a control. Both untreated (Figure 4A, left panel) and treated (Figure 4A, central and right panel) MCF7 cells were evaluated by flow cytometry for the quantification of the cells into the various phases of the cell cycle. Percentages of cells treated with empty liposome did not show statistically significant changes compared to untreated ones. Instead, treatment with Lip-NCBG halved the percentage of cells in the S phase and increased the number of cells in the G0/G1 phase in comparison to the MCF7 cells treated with Lip (Figure 4B). No significant change was detected in the G2/M phase between Lip and Lip-NCBG treated cells. Therefore, these data demonstrated an inhibitory impact of Lip-NCBG on the cell cycle progression of MCF7 cells.

### 2.6. Impact of Glo2 Inhibition on Doxorubicin (DOX) Cytotoxicity

**Figure 5:**
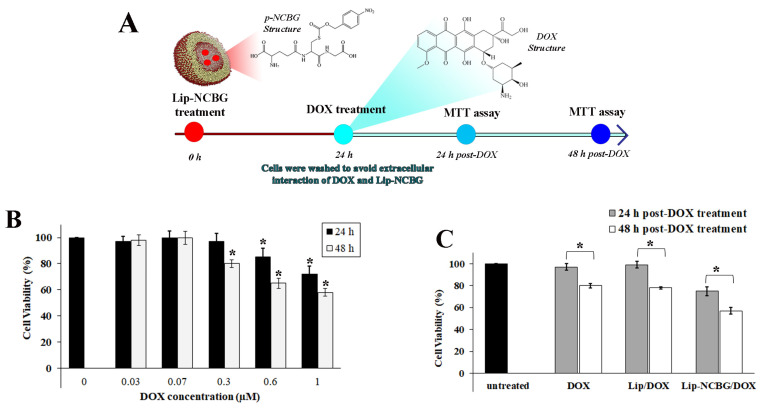
Cytotoxicity effect of combined treatment of p-NCBG inhibitor (Lip-NCBG) plus doxorubicin (DOX) on MCF7 cells. (**A**) Schematic diagram of combined Lip-NCBG/DOX administration. Cells were pre-treated with Lip-NCBG at final concentrations of 0.28 mM liposome and 0.25 mM p-NCBG (Figure S4A), for 24 h. Empty liposome (Lip) was used as control. Afterwards, the medium with Lip or Lip-NCBG was removed and cells were washed and treated with fresh medium with or without DOX at final concentration of 0.3 µM. MTT assay was performed after 24 and 48 h post-DOX treatment, (**B**) effect of different concentrations of DOX after 24 and 48 h and (**C**) effect of DOX, empty liposome plus DOX treatment (Lip/DOX) and liposome loaded with p-NCBG inhibitor plus DOX treatment (Lip-NCBG/DOX) after 24 and 48 h post-DOX treatment. The data are the mean of three experiments performed in triplicate ± SD. * *p* < 0.05.

To investigate the additive effect of Glo2 inhibition with a chemotherapeutic drug, we evaluated MCF7 cytotoxicity upon treatment with the doxorubicin (DOX), a commonly used anthracycline-class chemotherapy drug for treating breast cancer [27], testing six different concentrations for 24 and 48 h (Figure 5A). In line with the literature [28], DOX reduced MCF7 cell viability in a dose-dependent manner, resulting in 20 to 40% cell death after 48 h at the highest concentrations (0.3, 0.6 and 1 µM) (Figure 5B). Based on these results, we investigated the effect of Glo2 inhibition in combination with DOX on MCF7 cell viability by MTT assay. As for the cell cycle analyses, the safer and more effective concentration of Lip-NCBG was used, with the empty liposome being used as a control. Afterwards, the medium with Lip or Lip-NCBG was removed and cells were washed and treated with fresh medium containing or without DOX. An MTT assay was performed after 24 and 48 h post-DOX treatment (Figure 5A). Results revealed that the combined treatment of Lip-NCBG plus DOX significantly potentiated the cytotoxic effect of DOX at both times analyzed (Figure 5C).

We previously determined the DOX cytotoxicity in the HDF cells was showing an IC_50_ of 14 µM [29]. This is significantly higher than that observed in the MCF7 cells (IC_50_, 1.8 µM). Considering this DOX selectivity against the MCF7 cells with respect to HDFs, and the need to use these high concentrations, a potential synergy or additive effect between DOX and Lip-NCBG would be difficult to evaluate and would not be significant in any case.

### 2.7. Protein S-Glutathionylation in Relation to Glo2 Nuclear Presence

**Figure 6:**
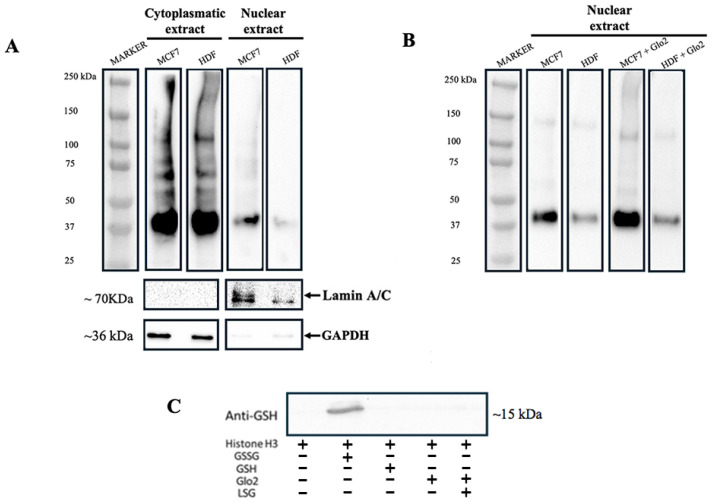
S-glutathionylation in MCF7 and HDF cells and the effect of exogenous incubation of recombinant Glyoxalase 2 (Glo2) on nuclear extracts. (**A**) Western blotting with anti-GS-P antibody of MCF7 and HDF cytoplasmic and nuclear extracts shows the S-glutathionylation profile of protein extracts exhibiting multiple bands corresponding to proteins undergoing S-glutathionylation. (**B**) Western blotting with anti-Glutathione antibody of MCF7 and HDF nuclear extracts non-incubated and incubated with exogenous recombinant Glo2 protein. In MCF7 nuclear extracts incubated with recombinant Glo2 there is an increase in S-glutathionylation. (**C**) In vitro S-glutathionylation of histone H3 subjected to different treatments. GSSG was used as a positive control and, indeed, shows S-glutathionylation on histone H3. No S-glutathionylation is evidenced with addition of GSH, recombinant Glo2 or even recombinant Glo2 with its substrate S-D-lactoylglutathione (SLG).

To investigate the involvement of Glo2 in protein S-glutathionylation, we analyzed protein S-glutathionylation levels in cytoplasmic and nuclear extracts of HDF and MCF7 cells and subsequently evaluated the role of Glo2. Figure 6 shows the levels of S-glutathionylation in cytosolic and nuclear extracts of MCF7 and HDF cells incubated with anti-GS-P antibody, which recognizes glutathionylated proteins. The pattern of S-glutathionylation in the cytoplasm of the HDF and MCF7 cells appears to be very similar both qualitatively and quantitatively, while the MCF7 nuclei show significantly more marked levels of S-glutathionylation than those of the HDF cells (Figure 6A). Since Glo2 S-glutathionylates cytoplasmic and mitochondrial target proteins, we evaluated the possibility for Glo2 to also S-glutathionylate nuclear proteins when it is located inside the nuclear compartment. Therefore, we incubated the nuclear extracts of MCF7 and HDF cells with recombinant human cytosolic Glo2 and subsequently evaluated the S-glutathionylation levels. Figure 6B shows a very marked increase in the level of S-glutathionylation when the nuclear extracts were incubated with recombinant Glo2, particularly in MCF7 cells compared to HDF cells. These data suggest an involvement of Glo2 in the S-glutathionylation of nuclear proteins and also highlight a substantial difference between cancerous and non-cancerous cells in relation to this regulatory PTM.

It has been shown that histone H3, among other post-translational modifications, is the one that can undergo S-glutathionylation [30]. Therefore, we attempted to explore whether Glo2 could have an S-glutathionylation effect on histone H3. So, we incubated histone H3 protein with recombinant Glo2 and evaluated S-glutathionylation by Western blotting. Unfortunately, the data show no interaction at the post-translational modification level between Glo2 and histone H3 (Figure 6C).

### 2.8. Glo2 Is Overexpressed in Different Cancer Cell Lines

**Figure 7:**
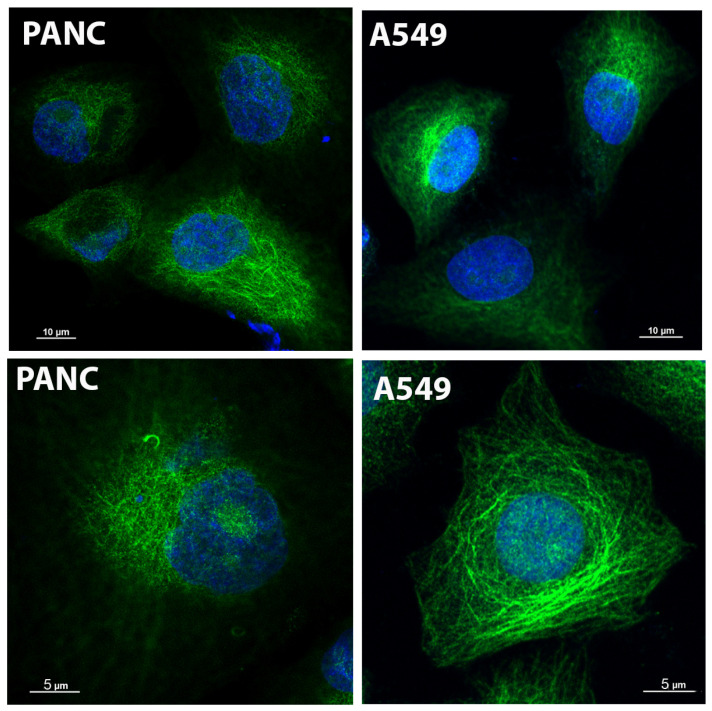
Immunofluorescence by confocal imaging of Glyoxalase 2 (Glo2) in different cancer cell lines. Immunofluorescence of Glo2 (green) in human pancreatic (PANC-1) and non-small cell lung cancer (A549) cell lines. The images were acquired at different magnifications to provide a representative view of cytoplasm (top row) and nucleus (blue) at single cell level (bottom row).

Glo2 is a ubiquitous enzyme, frequently overexpressed in several human cancers in addition to breast cancer [14,15,31,32]. To study whether additional cancer cell lines show Glo2 at nuclear level, we also performed Glo2 IF analyses in pancreatic carcinoma-1 (PANC-1) and non-small cell lung (A549) cancer cells (Figure 7). IF staining showed a marked increase in Glo2 expression both in the cytoplasm and nucleus of all cancer cell lines compared to the control HDF ones (Figure 1C). Moreover, altogether, these results suggest that a Glo2 overexpression pattern is common to cancer cells, at least in these cancer cell lines that we checked, and may open new possibilities for investigation in order to understand the role of Glo2 when it is recruited in the nucleus of some cancer cells.

### 2.9. Glo2 Isoforms’ Sequence Alignment and Prediction of Subcellular Localization Sites

**Figure 8:**
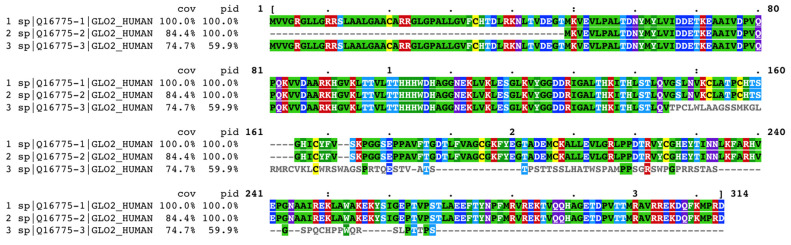
Clustal Omega multiple sequence alignment of human Glyoxalase 2 (Glo2) isoforms. Reference sequence is mitochondrial Glo2 (sp|Q16775-1|GLO2_HUMAN). Results are shown in Clustal w/Numbers output format, colored by identity, extracted and formatted by MView. Coloring method for protein alignment is in the Rasmol Amino Acid Colors. • positions with a single, fully conserved residue.

In UniProt Knowledgebase, human Glo2 is found as entry Q16775, with the name related to its function, a hydroxyacylglutathione hydrolase. The mitochondrial form has been chosen as the canonical sequence for the Q16775 entry but, in the sequence section, all Glo2 isoforms are present as sub-entries (Q16775-1, Q16775-2 and Q16775-3). Alternative splicing and alternative initiation, as described in the introduction, produce these isoforms. Q16775-1 is the sequence of the isoform 1 (the mitochondrial Glo2); it consists of 308 a.a. with a molecular mass of 33,806 Daltons. The first 48 amino acids in this sequence affect subcellular targeting as they contain an MTS, detected also in other vertebrates [22]. Q16775-2 is the sequence of the isoform 2 (the cytosolic form); it consists of 260 a.a. with a molecular mass of 28,860 Daltons. Q16775-3 is the sequence of isoform 3. It differs from the canonical form of isoform 1 in the 146-236 a.a. portion and lacks the 237–308 segment. This isoform has never been either isolated or crystallized but was identified by sequencing human cDNA [33]. Figure 8 illustrates the multiple sequence alignment of the three isoforms of Glo2 generated by Clustal Omega [34]. The reference sequence for the alignment has been assigned to Q16775-1, the mitochondrial form, as it represents the canonical sequence in the UniProt Knowledgebase. The percent coverage (cov) and percent identity (pid) of Q16775-2 and Q16775-3 with respect to the reference sequence Q16775-1 are calculated by MView [35], a utility included in the EMBL-EBI server that extracts and reformats the results of the multiple alignment. Figure 8 shows that Q16775-2, the cytosolic form, has 100% percent identity with 84.4% percent coverage compared to the mitochondrial form, the latter value being only due to the absence of the first 48 amino acids required for mitochondrial targeting. On the other hand, Q16775-3 has 59.9% percent identity and 74.4% percent coverage, retaining the first 48 amino acids and lacking the last 237–308 segment as well as there being differences in amino acids’ composition within the 146–236 range. Considering these characteristics, the mitochondrial (Q16775-1) and the cytosolic (Q16775-2) forms are identical; the only difference between them resides in the presence of the MTS in Q16775-1. On the other hand, the third isoform (Q16775-3) is quite different from the previous two and made us think about the possibility that this isoform could localize elsewhere, even though it has the mitochondrial targeting sequence as Q16775-1. In fact, it is not uncommon that several mitochondrial proteins exhibit nuclear localization as a result of cellular and environmental stimuli [36]. Among the various mitochondrial proteins that play a nuclear role we find, for example, TFAM. This is present in species such as rats, mice and humans; in the nucleus it carries out the transcription functions of nuclear genes and promotes cytoprotection against chemotherapeutic drugs. The stimulus driving nuclear localization is steady-state conditions [37,38,39].

Considering this, we decided to submit the Q16775-3 sequence to PSORT II to predict a possible alternative localization of this isoform compared to those already known (Q16775-1 and Q16775-2). As can be seen in Table 3, the results obtained by PSORT II show a correct prediction for mitochondrial (Q16775-1) and for cytosolic (Q16775-2) forms by the k-NN method. In addition, both forms result in cytoplasmic proteins in the NNCN score, as it discriminates only in favor of the tendency to be nuclear or cytoplasmic proteins, therefore, the presence of the classical type of NLSs (pat4, pat7) or bipartite NLS is obviously absent [40]. With regard to Q16775-3, interesting results are shown in Table 3 as PSORT II predicts a nuclear localization both by the k-NN method and the NNCN score for this isoform, but no NLSs have been detected. If the first 48 amino acids that contain an MTS are removed from the Q16775-3 sequence and then submitted again to PSORT II, the results show that the prediction of nuclear localization greatly increases not only for the k-NN method (from 39.1% to 73.9%) but also for the NCNN score (increasing from 55.5 to 76.7 in reliability). These results show that for the Q16775-3 sequence a nuclear localization prediction is made by PSORT II, but also highlight that the differences in amino acids’ composition within the Q16775-1 sequence are possibly due to a different subcellular localization.

It must be emphasized, however, that the immunogen for the anti-Glo2 antibody used in our experiments is directed towards the C terminal of Glo2 within the region STLAEEFTYNPFMRVREKTVQQHAGETDPVTTMRAVRREKDQFKMPRD. This region is present only in Q16775-1 and Q16775-2 sequences, therefore, with this commercial anti-Glo2 antibody, only mitochondrial (Q16775-1) and cytosolic (Q16775-2) forms are detected. In this scenario, after bioinformatic analysis, we can, therefore, certainly assert that the Glo2 protein is not structurally addressed in the nucleus because of the absence of the NLS, whereas it is possible to speculate that it is a transient protein that dynamically moves into the nucleus only under certain cell conditions. We do not exclude the possibility that it may be the mitochondrial form that migrates to the nucleus through a pathway associated with specific events in the mitochondria, as seen for several proteins that contain an MTS, and then translocates into the nucleus when mitochondria signal their status to the cell [41].

## 3. Discussion

In this study, we evaluated the Glo2 enzyme in two different cell lines: a cancer cell line (MCF7) and a non-tumor cell line (HDF), analyzing its overexpression and its possible nuclear localization in relation to cell proliferation. In addition, we examined the effect of Glo2 inhibition in combination with doxorubicin, a drug commonly used in breast cancer chemotherapy. Taken together, the results of this study suggest a possible role for Glo2 in conferring resistance and promoting the survival of breast cancer cells. In this study, for the first time, Glo2 was localized in the nucleus, beyond the usual presence found in the cytoplasm and mitochondria. Furthermore, a greater presence of Glo2 was detected in the cytoplasm and nucleus compartments of cancer cells than in non-tumor cells, suggesting a possible specific involvement of this protein in cancer cells.

Specifically, the study was conducted on MCF7 tumor cells, which showed overexpression of Glo2 in both Western blotting and immunofluorescence analyses. Additionally, confocal microscope images showed overexpression of Glo2 in both the cytosolic and nuclear compartments in two other tumor cell lines (PANC and A549). These results suggest that the high amount of Glo2 may be a distinguishing feature of tumor cells, correlated with increased survival resistance and proliferative capacity.

Indeed, numerous studies have demonstrated that Glo2 is overexpressed in cancer cells. This overexpression is attributed to the fact that the high glycolytic flux of cancer cells produces elevated levels of methylglyoxal (MGO), a toxic by-product. The glyoxalase system, including Glo2, plays a crucial role in rapidly metabolizing MGO as its accumulation can trigger apoptosis, leading to the rapid death of cancer cells. Several studies have also suggested that Glo2 may function as a survival factor through mechanisms involving survival pathways that are more active in cancer cells [11,12,13,14,15]. Therefore, we hypothesize that Glo2 has an additional role related to cell survival, potentially linked to its presence in the nucleus. In our study, the data obtained from Western blotting of cytoplasmic extracts reveal a double band: one corresponding to the canonical weight of Glo2 at 29 kDa and another band at 47 kDa. However, at the nuclear level, we can observe that the 47 kDa band is distinctly much more intense in the nucleus of MCF7 cells than in HDF cells. This significant difference suggests that Glo2 may exist within the nucleus in a form that supports functions beyond those commonly associated with the glyoxalase system. The experiment conducted with incremental additions of DTT indicates that the 47 kDa band results from the interactions between Glo2 and other proteins that may include isoforms of Glo2 itself. Notably, the 47 kDa band persisted even after treatment with increasing concentrations of NaCl and was predominantly found in the second step of chromatin extraction, suggesting a possible interaction with non-histone proteins within the nucleus of cancer cells. Anyway, the significantly greater presence of Glo2 in MCF7 tumor cells compared to non-tumor HDF cells suggests a potential involvement of Glo2 in the regulation of cell proliferation and resistance to chemotherapeutic treatments. To investigate this, we performed cell cycle regulation studies. Analysis of Glo2’s presence across different phases of the cell cycle revealed a correlation between its expression and the proliferative phases of the cells. Specifically, the amount of Glo2 significantly increases during the S and G2/M phases of the cell cycle, more markedly in MCF7 cells. This increase in Glo2 during proliferative phases suggests that Glo2 may play a role in facilitating cell proliferation in cancer cells. Glo2 enzyme activity was measured in both cytosolic and nuclear extracts during the G0/G1 and G2/M phases. The data indicate that there is not a direct correlation between the amount of protein and its enzyme activity. This is not surprising as previous studies have confirmed that Glo2 operates several post-translational modifications (PTMs), including S-glutathionylation, on target proteins and these modifications can sometimes limit its enzymatic activity [6,7]. We can, therefore, speculate that Glo2 may interact with specific proteins through PTMs, potentially playing a role in the regulation of cancer cell proliferation and survival. Indeed, in our experiments, we found that nuclear extracts of MCF7s showed a more robust pattern of S-glutathionylation than nuclear extracts of non-tumor cells. Interestingly, when pure recombinant Glo2 was added to cytoplasmic or nuclear extracts it significantly increased S-glutathionylation, particularly in tumor cells. This finding indicates a direct involvement of Glo2 in the S-glutathionylation of nuclear and cytoplasmatic proteins, and, moreover, highlights a substantial difference between tumor and non-tumor cells in relation to the regulatory effect of PTMs.

To evaluate the effect of Glo2 on proliferation regulation, we treated cells with a specific Glo2 inhibitor, p-nitrocarbobenzoxyglutathione (p-NCBG). The results obtained are surprising as MCF7 cells, after treatment with Glo2 inhibitor, showed cell cycle inhibition, demonstrating an effective involvement of Glo2 with proliferation control (Figure 4). To evaluate a possible use of the inhibitor as an additive in chemotherapy, we assessed the cytotoxicity effect of the inhibitor with doxorubicin treatment. Doxorubicin is a widely used chemotherapeutic agent in the treatment of breast cancer. However, many cancer cells develop resistance to this drug, thus, reducing its efficacy. In this context, Glo2 inhibition has been investigated as a possible strategy to increase the sensitivity of cancer cells to doxorubicin. The fact that the synthetic Glo2 inhibitor, p-nitrocarbobenzoxyglutathione (p-NCBG), increases the cytotoxicity of doxorubicin in MCF7 cells further supports the idea that Glo2 may act as a survival factor for breast cancer cells. This result is particularly interesting because it suggests that the combination of Glo2 inhibitors with traditional chemotherapeutic agents could be an effective therapeutic strategy. However, it can be speculated that the ability of the inhibitor to increase the effect of doxorubicin may be related to an increase in oxidative stress due to Glo2 inhibition. This enhanced oxidative stress could lead to a decreased ability of cells to neutralize toxic alpha-oxaldehydes, thereby increasing the sensitivity of cancer cells to chemotherapeutic drugs such as doxorubicin. This synergistic effect suggests that Glo2 contributes to the resistance of cancer cells to chemotherapeutic treatments. When Glo2 is inhibited, tumor cells are less able to handle doxorubicin-induced oxidative stress and DNA damage, leading to increased cell death.

Our results are consistent with previous studies indicating an increased presence of Glo2 in cancer cells and, in some cases, attributing a specific antiapoptotic role to this protein. Our data confirm that Glo2 acts as a pro-survival protein, capable of increasing tumor cell resistance to chemotherapeutic treatments such as doxorubicin. Importantly, to date, there are fewer studies on Glo2 than on Glo1, which, instead, consistently show overexpression of Glo1 in different cancer cell lines. Taken together, these data suggest that the glyoxalase system is overexpressed in some cancer cells, providing an advantage for survival and resistance to apoptosis, key features of uncontrolled cancer proliferation [4,32]. Of particular interest is the demonstration of the presence of Glo2 in the nucleus. Considering the presence of two isoforms, one cytosolic and one mitochondrial, it is likely that Glo2 is found in the nucleus in a transient form related to proliferation-associated cellular activities. This hypothesis is supported by the fact that nuclear Glo2 is significantly more present in cancer cells with a high proliferative rate.

## 4. Materials and Methods

### 4.1. Cell Culture and Nuclear Extracts

MCF7s (mammary adenocarcinoma epithelial cells, HTB-22), HDFs (human skin fibroblasts, PCS-201-012), PANC-1 (pancreatic carcinoma epithelial cells, CRL-1469) and A549 (lung carcinoma epithelial cells, CRM-CCL-185) were purchased from ATCC (American Type Culture Collection, Manassas, VA, USA). Cells were cultured in DMEM medium (11965092, Gibco, Grand Island, NY, USA), supplemented with 10% (*v*/*v*) heat-inactivated fetal calf serum (A5256701, Gibco, Grand Island, NY, USA), 2 mM glutamine (25-005-CI, Corning, Glendale, AZ, USA), 10 U/mL penicillin (30-001-CI, Corning, Glendale, AZ, USA) and 10 mg/mL streptomycin (30-001-CI, Corning, Glendale, AZ, USA) at 37 °C in a humidified atmosphere containing 5% (*v*/*v*) CO_2_. Nuclear extracts were obtained using the nuclear extraction kit (78833, Thermo Fisher Scientific, Waltham, MA, USA) according to the manufacturer’s instructions, and quantified with the Bradford assay (5000205, Bio-Rad, Hercules, CA, USA) by spectrophotometer at 595 nm of absorbance.

### 4.2. Preparation of Recombinant Glo2

Recombinant human cytosolic Glo2 was prepared by heterologous expression in *Escherichia coli* from a human adult liver complementary DNA library and subsequently purified in Affi-Gel 10 (Bio-Rad), as previously reported by Ridderstrom et al. [21]. Fraction positives for Glo2 activity were pooled. Purity of the enzyme was evaluated by sodium dodecyl sulfate-polyacrylamide gel electrophoresis (SDS-PAGE) and was close to 98%. Protein concentration was determined by the method of Bradford [42] using bovine serum albumin (BSA) as standard.

### 4.3. SDS-PAGE and Western Blotting

An amount of 20 µg of nuclear and cytoplasmatic extracts for each sample were used and Western blotting was performed under reducing conditions with 25 mM DTT (10197777001, Sigma-Aldrich, Saint Louis, MO, USA), except for protein S-glutathionylation and sequential salt extraction assays, which were performed in non-reducing conditions. Primary antibodies used were the following: Glo2 (rabbit polyclonal, 1:500, TA334983, OriGene, Rockville, MD, USA), glutathione-protein complex (mouse monoclonal, 1:1000, sc-52399, Santa Cruz Biotechnology, Dallas, TX, USA), lamin C/A (rabbit polyclonal, 1:1000, 2032, Cell Signaling Technology, Beverly, MA, USA) and GAPDH (rabbit monoclonal, 1:2000, MA5-35235, Invitrogen, Carlsbad, CA, USA). Signal was detected using horseradish peroxidase (HRP)-linked goat anti-mouse (polyclonal, 1:5000, sc-2005, Santa Cruz Biotechnology) or goat anti-rabbit (polyclonal, 1:5000, TA130015, OriGene, Rockville, MD, USA) secondary antibodies. Immunoblots were detected by chemiluminescence using Chemidoc Instrument (Bio-Rad, Hercules, CA, USA). Complete images with the ladder with weight references were obtained using the Merge function of the program Image Lab 6.1 (Bio-Rad, Hercules, CA, USA). The Image Lab 6.1 program is directly connected to the ChemiDoc used for our Western blotting, which is programmed to automatically expose the markers on each loaded membrane. Densitometry quantification of the immunoblot was performed using Image Lab 6.1 software (Bio-Rad, Hercules, CA, USA). To quantify Western blot bands with Image Lab 6.1, the images have been imported into the software and ”Auto Lane Detection” function was used to automatically detect lanes. Subsequently, the ”Detect Bands” command was used to identify the bands, thus, displaying the intensity of the bands in the ”Analysis Table”. At the end, the data were normalized using a housekeeping gene (e.g., GAPDH). Densitometry quantification of the immunoblot was performed using Image Lab software (Bio-Rad, Hercules, CA, USA). To check for non-specific signals, the anti-Glo2 antibody was first incubated with recombinant Glo2 so that the Glo2 binding sites on the antibody could be saturated. Subsequently, the membrane was incubated with this antibody.

### 4.4. Immunofluorescence and Confocal Microscopy Imaging

Immunofluorescence (IF) was performed on MCF7s and HDFs according to standard protocols; cells were visualized on a Nikon-AR1 confocal microscope. Cells were fixed in 4% paraformaldehyde, permeabilization was performed with 0.1% Triton X-100 (X100, Sigma-Aldrich, Saint Louis, MO, USA) and cells were incubated overnight at 4 °C with anti-Glo2 antibody (rabbit polyclonal, 1:500, TA334983, OriGene, Rockville, MD, USA). Cells were then incubated with Alexa Fluor 488-conjugated goat anti-rabbit secondary antibody (polyclonal, 1:400, A-11008, ThermoFisher Scientific, Waltham, MA, USA) for 30 min at room temperature. Finally, the slides were cover-slipped with Prolong glass antifade mountant with NucBlue stain. Negative controls were performed by omitting the primary antibody.

### 4.5. Enzymatic Activities

Glo2 activity was analyzed as previously described by Principato et al. [43]. Briefly, samples were incubated in a reaction mixture containing 100 mM MOPS buffer pH 7.2, 0.8 mM SLG and 0.2 mM DTNB at 25 °C. Reactions were assayed by monitoring the absorbance increase at 412 nm (ε = 13.6 mM^−1^ cm^−1^). One enzyme unit (U) is defined as the amount of enzyme catalyzing the hydrolysis of 1 µmol of SLG per minute at a saturating substrate concentration.

### 4.6. Sequential Salt Extraction Assay

Sequential salt extraction assay was performed as previously described [43]. Briefly, cultured cells were resuspended in hypotonic buffer A (0.3 M sucrose, 60 mM KCl, 60 mM Tris pH 8.0, 2 mM EDTA and 0.5% NP-40) and centrifuged at 6000× *g* for 10 min at 4 °C to obtain the nuclear pellet. Six salt concentrations (0, 100, 200, 300, 400 and 500 mM NaCl) were prepared using 1X mRIPA solution (100 mM Tris pH 8.0, 2% NP-40 and 0.5% sodium deoxycholate) plus protease inhibitors (539136, Sigma-Aldrich, Saint Louis, MO, USA). The nuclear pellets were resuspended in increasing concentrations of NaCl to isolate the different chromatin fractions. Each fraction was analyzed for Glo2 quantification by Western blotting. Equivalent volumes of lysate, rather than equivalent protein concentrations, were loaded into SDS acrylamide gel to evaluate protein binding profile [44].

### 4.7. Cell Cycle Analysis

Starvation was induced with DMEM (11965092, Gibco, Grand Island, NY, USA) containing 0.1% FBS (A5256701, Gibco, Grand Island, NY, USA) [25]. After 72 h, cells were subsequently reactivated with DMEM supplemented with 15% FBS. Cells were harvested at time 0 and after 48 h from reactivation. Cell cycle phases were determined using the fluorescent DNA dye propidium iodide (40 μg/mL) (P4170, Sigma-Aldrich, Saint Louis, MO, USA) and DNase-free RNaseA (100 mg/mL) (10109142001, Sigma-Aldrich, Saint Louis, MO, USA). All cells were analyzed using a Guava EasyCyte flow cytometer (MilliporeSigma, Burlington, NJ, USA). Briefly, cells resuspended in cold PBS (10010023, Gibco, Grand Island, NY, USA) were slowly added to ice cold 70% ethanol by vortexing and incubated on ice for 45 min. Cells were then centrifuged for 10 min at 4 °C at 1200 rpm. Supernatant was aspirated and cell pellet was suspended in a master mix solution containing PBS, propidium iodide and DNase-free RNaseA for 30 min at room temperature in the dark. At least 10,000 cells for each sample were measured. Percentages of cells into different cycle phases (G0/G1, S and G2/M) were quantified by MultiCycle AV DNA analysis plug-in for FCS Express (De Novo Software, Version 6.06).

### 4.8. Carbobenzoxyglutathione (CBG) Synthesis and Liposomes Preparation

Synthesis of p-nitrocarbobenzoxyglutathione (p-NCBG) was performed as previously described [45]. The ability of p-NCBG was determined by measuring the inhibition level of the enzyme activity “in vitro” in presence of increasing inhibitor concentrations and comparing with Glo2 enzymatic activity measured in the absence of p-NCBG. Empty (control, Lip) and p-NCBG-containing liposomes (Lip-NCBG) were prepared by “thin film hydration” method, as previously described [26]. Chloroform solutions of 1-palmitoyl-2-oleoyl-glycero-3-phosphocholine (POPC) were added to rounded bottom flask; then, after removing the solvent by evaporation under reduced pressure, the film was dried under vacuum for 4 h and then resuspended in the required amount of p-NCBG dissolved in MOPS (A2947, Applichem GmbH, Darmstadt, Germany) 100 mM buffer (pH 7.2) to obtain Lip-CBG. The final POPC and CBG concentrations were 3 mM and 2.5 mM, respectively. After liposome preparation, MCF7 cells were treated with appropriate amounts of free p-NCBG (p-NCBG) and liposome-encapsulated p-NCBG (Lip-NCBG) for 6, 12, and 24 h to obtain final p-NCBG concentrations of 0.12, 0.25 and 0.35 mM. After having obtained the pellets of the treated cell, we proceed with the protein extraction. Finally, the ability of p-NCBG to inhibit cellular Glo2 was determined on cell extracts by measuring the enzyme activity of Glo2 and comparing it to the activity measured in untreated cells.

### 4.9. Cytotoxicity Tests

MCF7 cells were seeded in 24-well plates at 4 × 10^4^ cells/well to reach 60% of confluence after 24 h. Then, cells were incubated with empty liposomes (Lip) or 0.3 mM Glo2 inhibitor-containing liposomes (Lip-NCBG) for 24 h, and subsequently washed with PBS (Gibco, Grand Island, NY, USA) to remove the liposomes eventually present in medium. Finally, MCF7 cells were treated with the chemotherapy drug doxorubicin (Dox) at the final concentration of 0.25 µM for 24 h. Previous cell viability tests on MCF7 cells were performed to establish the safe doses of Lip, Lip-NCBG and Dox after 48 h of treatment. Cytotoxicity was determined by 3-(4,5-dimethylthiazol-2-yl)-2,5-diphenyltetrazolium bromide (MTT) (M2128-1G, Sigma-Aldrich, Saint Louis, MO, USA) assay following a well-established procedure [46]. The absorbance of purple formazan crystal, produced by reduction in MTT reactive, was read on a multiwell scanning microplate reader (BioTek Synergy HT MicroPlate Reader Spectrophotometer, BioTek Instruments Inc., Winooski, VT, USA) at 570 nm using the extraction buffer as a blank. The optical density in the control group (untreated cells) was considered as 100% viability. The relative cell viability (%) was calculated as (A570 of treated samples/A570 of untreated samples) × 100. Determinations were carried out in triplicate in each experiment and mean ± S.D. from five independent experiments was calculated.

### 4.10. Statistical Analysis

The overall data obtained are from at least four experiments and are shown as mean ± S.D. Statistical comparison of the differences among groups of data was carried out using Student’s *t*-test. We considered all *p* values ≤ 0.05 significant and *p* values ≤ 0.01 highly significant.

### 4.11. Multiple Sequence Alignment

Alignment of the three isoforms of Glo2 has been generated by submitting the sequences of the three isoforms of Glo2 available in UniProt Knowledgebase at Q16775 entry (https://www.uniprot.org, accessed on 11 March 2024) to the EMBL’s European Bioinformatics Institute (EMBL-EBI) sequence analysis service web server using the multiple sequence alignment tool Clustal Omega (URL https://www.ebi.ac.uk/jdispatcher/msa/clustalo, accessed on 12 March 2024) [47]. Clustal Omega is a multiple sequence alignment program that uses seeded guide trees and HMM profile-profile techniques to generate alignments between three or more sequences.

### 4.12. Prediction of Subcellular Localization Sites

To predict the localization of Glo2 isoforms, a free Internet computer program that is widely used to predict the subcellular localization sites of proteins from their amino acid sequences has been used. This computer program is PSORT II (URL https://psort.hgc.jp/form2.html, accessed on 19 March 2024): it receives the information from an amino acid sequence and its source origin and analyzes the input sequence by applying the stored rules for various sequence features of known protein sorting signals. PSORT II program was developed by Dr. Nakai’s group (Human Genome Center, Institute for Medical Science, University of Tokyo, Japan) using the k-nearest neighbor (k-NN) method [48]. The k-nearest neighbor method is a pattern-recognition algorithm in which k is a predefined parameter. The prediction is performed using the k-nearest data points, where k is a predefined integer parameter. If these k data points contain, say, cytosolic proteins with 50%, the query is predicted to be localized to the cytosol with the probability of 50% [49]. For the recognition of nuclear proteins, PSORT II performs a search of potential nuclear localization signal (NLS) sequences but uses also a score (called “NNCN”) that discriminates the tendency to be at either the nucleus or the cytoplasm based on the amino acid composition according to the neural network constructed by Reinhardt [50].

## 5. Conclusions

In summary, the collected data indicate that Glo2 plays a crucial role in the survival and proliferation of breast cancer cells, likely through specific interactions with cytoplasmic and nuclear proteins and probably involving PTMs such as S-glutathionylation. These interactions justify further investigation in future studies. Glo2 inhibition could represent a promising therapeutic strategy to enhance the efficacy of existing chemotherapeutic agents, such as doxorubicin. These findings increase knowledge about Glo2 [51] and open new perspectives for the development of targeted therapies against breast cancer and other cancers that express high levels of Glo2, although further research is needed to investigate the additional role of Glo2 in cancer.

## Figures and Tables

**Table 1:** Cell synchronization data of MCF7 and HDF cell lines. The table represents the proliferation rates in the different phases of the cell cycle of both lines. The synchronized cells at time 0 are predominantly in the G0/G1 phase (non-proliferative phase), whereas at time 48 both cell lines showed a greater prevalence in the G2/M phase, particularly the MCF7 cell line.

	G0/G1	S	G2/M
MCF7 synchronized at T0	61.2%	13.7%	22.6%
HDF synchronized at T0	81.7%	2.5%	10.4%
MCF7 synchronized at T48	46.9%	11.7%	35.5%
HDF synchronized at T48	52.1%	12.3%	26.8%

**Table 2:** p-NCBG inhibitory action. We pharmacologically inhibited Glyoxalase 2 (Glo2) by using p-nitrocarbobenzoxyglutathione (p-NCBG), which specifically inhibits mammalian Glo2 without affecting Glo1. Glo1 and Glo2 activity levels were tested at different concentrations (9, 18, 36 and 72 µM) of competitive inhibitor of mammalian Glo2 p-nitrocarbobenzoxyglutathione (p-NCBG). Glo1 and Glo2 activities are given as mU/mL. Results are reported as mean ± S.D. of at least *n* = 4 different experiments. * *p* ≤ 0.01.

p-NCBG Concentrations (μM)	Glo1 Activity (mU/mL)	Glo2 Activity (mU/mL)
0	48.1 ± 3.1	88.7 ± 5.3
9	48.4 ± 1.6	3.8 ± 0.7 *
18	47.2 ± 3.5	4.2 ± 1.1 *
36	46.3 ± 1.3	2.5 ± 0.5 *
72	45.2 ± 1.1	0.3 ± 0.01 *

**Table 3:** Prediction of subcellular localization of Glo2 isoforms’ sequences by PSORT II. NUCDISC: discrimination of nuclear localization signals; NNCN: Reinhardt’s method for cytoplasmic/nuclear discrimination and k-NN: k-nearest neighbor algorithm for assessing the probability of localizing protein subcellular locations (mit: mitochondrial; cyt: cytoplasmic and nuc: nuclear). Q16775-3 ** is Q16775-3 sequence with first 48 amino acids removed.

Input Sequence	NUCDISC	NNCN	k-NN
Q16775-1	pat4: none	cytoplasmic	39.1%: mitochondrial
	pat7: none	reliability: 94.1	34.8%: cytoplasmic
	bipartite: none		prediction is mit
Q16775-2	pat4: none	cytoplasmic	56.5%: cytoplasmic
	pat7: none	reliability: 94.1	21.7%: cytoskeletal
	bipartite: none		prediction is cyt
Q16775-3	pat4: none	nuclear	39.1%: nuclear
	pat7: none	reliability: 55.5	39.1%: mitochondrial
	bipartite: none		prediction is nuc
Q16775-3 **	pat4: none	nuclear	73.9%: nuclear
	pat7: none	reliability: 76.7	13.0%: cytoplasmic
	bipartite: none		prediction is nuc

## Data Availability

Data are contained within the article or Supplementary Material.

## Supplementary Materials

The following supporting information can be downloaded at: https://www.mdpi.com/article/10.3390/ijms252010888/s1.
