# Supplementary material for: Overexpression of Glyoxalase 2 in Human Breast Cancer Cells: Implications for Cell Proliferation and Doxorubicin Resistance
Source: Int J Mol Sci. 2024 Oct 10;25(20):10888. doi: 10.3390/ijms252010888 (PMC11507095; doi:10.3390/ijms252010888)
Supplement: Supplementary file 1 [file ijms-25-10888-s001.zip › ijms-3138282-supplementary.pdf]

Supporting Information

Overexpression of Glyoxalase 2 in Human Breast Cancer Cells: Implications for Cell Proliferation and Doxorubicin Resistance

Authors: Brenda Romaldi [1,†], Andrea Scirè [2,†], Cristina Minnelli [2], Andrea Frontini [2], Giulia Casari [1], Laura Cianfruglia [1], Giovanna Mobbili [2], Lidia de Bari [3], Cinzia Antognelli [4], Federico V. Pallardó [5,6] and Tatiana Armeni [1,*]

Corresponding Author: Tatiana Armeni, Department of Odontostomatologic and Specialized Clinical Sciences, Università Politecnica delle Marche, 60131 Ancona, Italy; t.armeni@staff.univpm.it

Table of Contents:

S1. Western blotting analysis for the evaluation of non-specific bands 2

S2. Cell cycle cytograms of HDF and MCF7 cells synchronized at T0 and T48 3

S3. Glo2 activity after treatment with free p-NCBG in MCF7 cells 3

S4. Cytotoxicity of Lip or Lip-NCBG at increasing administration after 48h of treatment 4

S5. Glo2 inhibition by p-NCBG in MCF7 cells 5

S6. Western blotting analysis with relative marker of MCF7 and HDF after NaCl treatment 5

S1. Western blotting analysis for the evaluation of non-specific bands

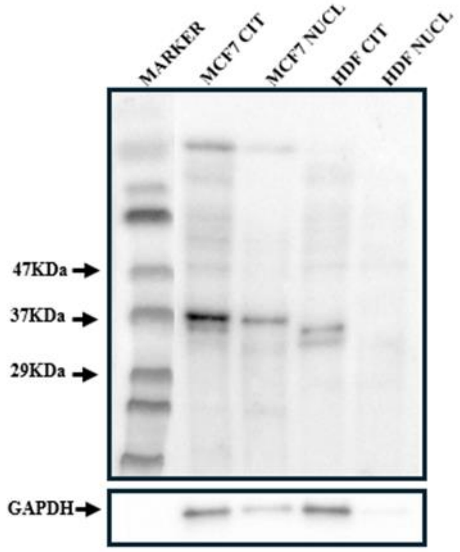

Figure S1. Western blotting analysis for the evaluation of non-specific bands. Representation of ChemiDoc images of cytosolic and nuclear extracts of MCF7 and HDF. Anti-Glo2 was previously incubated with recombinant Glo2 to block the antibody. Western blotting was performed with Anti-Glo2. Subsequently, membrane was developed at ChemiDoc (Bio-Rad Laboratories, California, USA). The membrane was incubated with anti-GAPDH antibody and developed at ChemiDoc. We can only observe the band at 37 kDa which results as an aspecific band, whereas the 29 and 47 KDa bands are not present.

S2. Cell cycle cytograms of HDF and MCF7 cells synchronized at T0 and T48

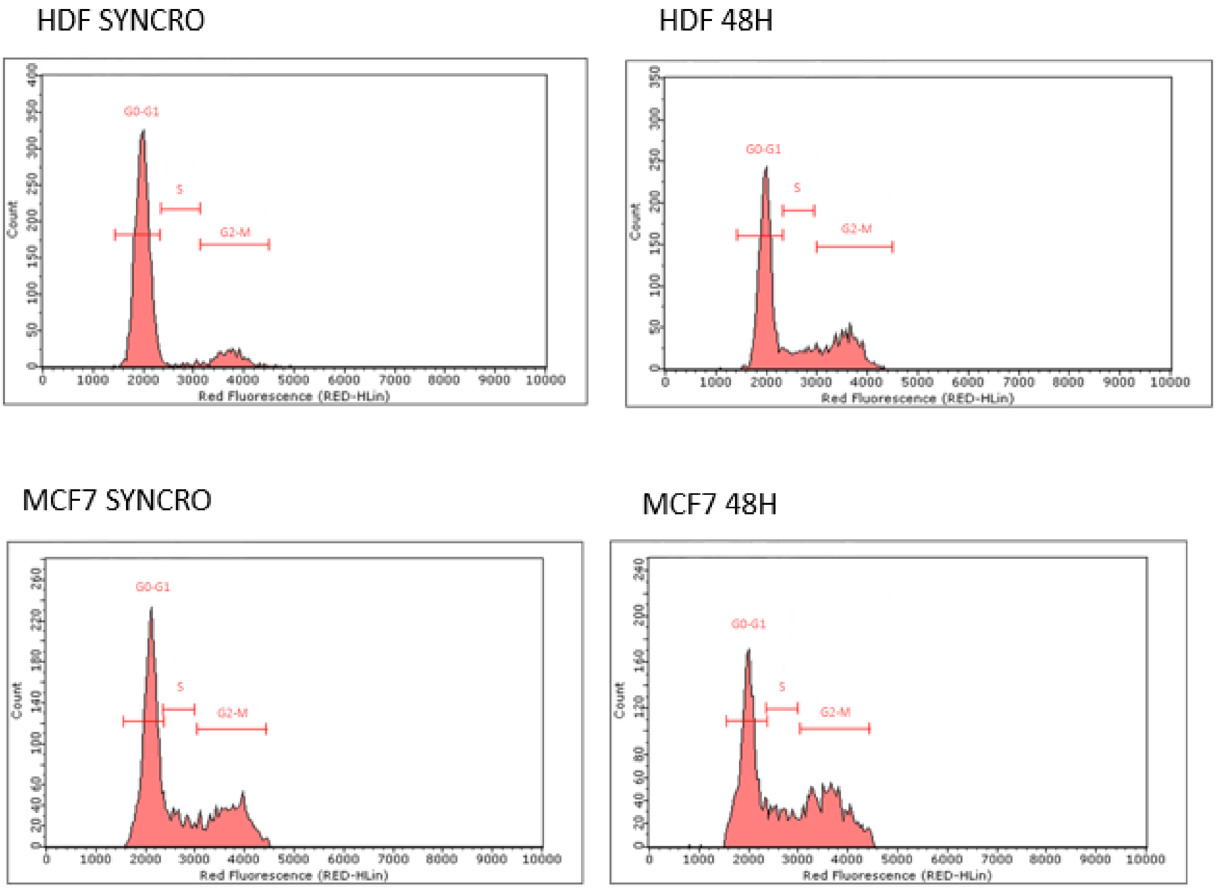

Figure S2. The figure shows the cell cycle cytograms of the HDF and MCF7 cell lines synchronized at time 0 (left panels) and after reactivation at time 48 (right panels). At time 0, an increase in the percentage of cells in G0/G1 phase was observed in both cell lines. Instead, 48 hours after reactivation, there was an increase in the percentage of cells in G2/M phase, with a particularly pronounced increase in MCF7 cells.

S3. Glo2 activity after treatment with free p-NCBG in MCF7 cells

Free p-NCBG concentration (µM)	Glo2 activity (nmol/min/mg prot) at 12h	Glo2 activity (nmol/min/mg prot) at 24h
0	0,15 ± 0,01	0,15 ± 0,02
100	0,14 ± 0,03	0,13 ± 0,01
200	0,14 ± 0,02	0,13 ± 0,02

Figure S3. MCF7 cells were treated with free p-NCBG (not encapsulated in liposomes) at concentrations of 100 and 200 µM. After 12 and 24 hours, the respective protein extracts

were obtained and quantified to evaluate Glo2 activity. No significant variation of Glo2 activity has been observed after treatment with free p-NCBG at both tested concentrations. The activity of Glo2 was calculated as described in the material and methods section.

S4. Cytotoxicity of Lip or Lip-NCBG at increasing administration after 48 h of treatment

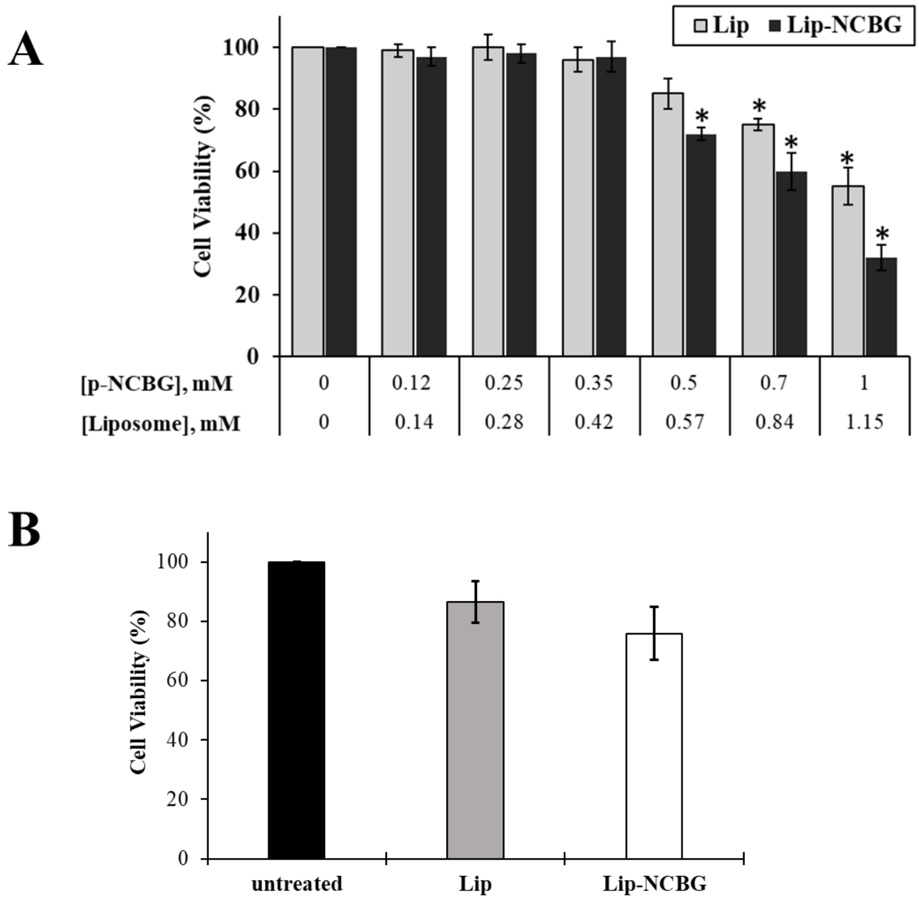

Figure S4. To identify the safe Lip-NCBG concentration, cytotoxicity studies were performed on MCF7 cells (A) using Lip-NCBG and Lip as control. Liposomes loaded with 0.25 mM of inhibitor, which showed no toxicity and about 60% of Glo2 activity inhibition, were used. HDF cell line was then treated with the selected concentration of Lip-NCBG and empty Lip to assess the safety of treatment in the normal cell line (B). *$P<0.05$, significative difference with respect to untreated cells.

S5. Glo2 inhibition by p-NCBG in MCF7 cells

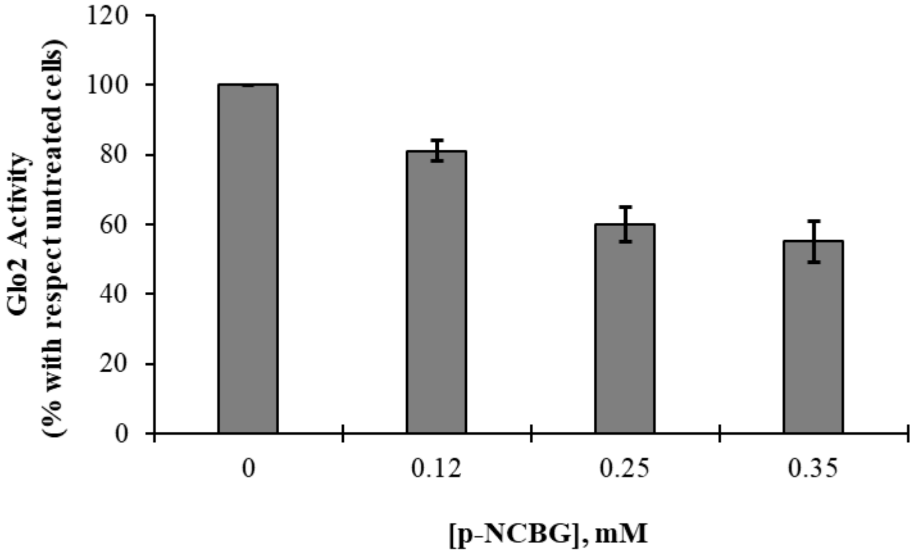

Figure S5. MCF7 cells were treated with increasing concentrations of liposomes loaded with p-NCBG in the range of 0.12-0.35 mM. After 24 hours, the cell pellets and the respective protein extracts were obtained. Protein extracts were quantified and used to evaluate Glo2 activity. Activity of Glo2 was calculated as described in the material and methods section.

S6. Western blotting analysis with relative marker of MCF7 and HDF cells after treatment with NaCl

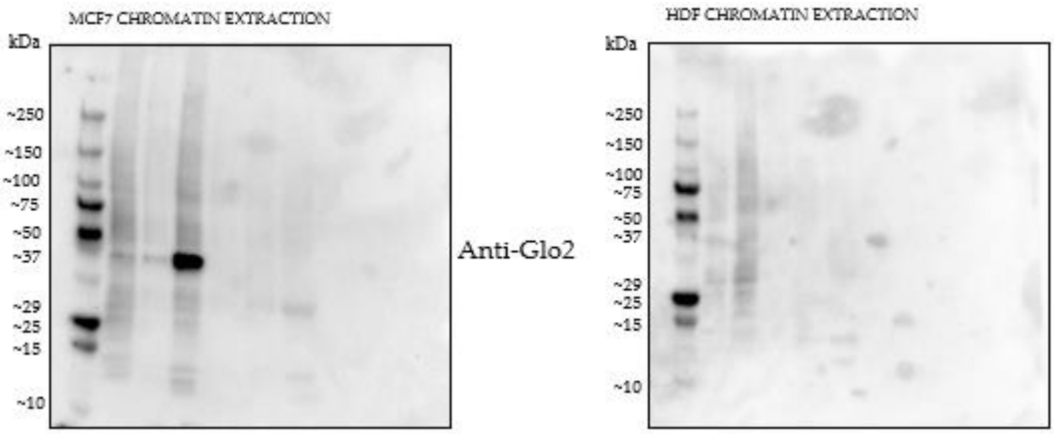

Figure S6. Sequential salt extraction on nuclei of MCF7 and HDF cells and immunoblot of Glo2. Left panel showed MCF7 chromatin extraction with relative marker and molecular weights,

whereas right panel displayed HDF chromatin extraction with relative marker and molecular weights.
